# High incidence and early onset of nivolumab-induced pneumonitis: four case reports and literature review

**DOI:** 10.1186/s12890-018-0592-x

**Published:** 2018-01-30

**Authors:** N. Koyama, O. Iwase, E. Nakashima, K. Kishida, T. Kondo, Y. Watanabe, H. Takahashi, Y. Umebayashi, Y. Ogawa, H. Miura

**Affiliations:** 1 Department of Clinical Oncology, Tokyo Medical University Hachioji Medical Center, Tokyo, 193-0998 Japan; 2 Department of Hematology, Tokyo Medical University Hachioji Medical Center, Tokyo, Japan; 3 Department of Thoracic Surgery, Tokyo Medical University Hachioji Medical Center, Tokyo, Japan; 4 Department of Dermatology, Tokyo Medical University Hachioji Medical Center, Tokyo, Japan; 5 Department of Otolaryngology-Head and Neck Surgery, Tokyo Medical University Hachioji Medical Center, Tokyo, Japan

**Keywords:** Drug-induced pneumonitis, Immune checkpoint inhibitor, Nivolumab, Non-small cell lung cancer, Malignant melanoma, Hypopharyngeal carcinoma

## Abstract

**Background:**

Nivolumab, an anti-programmed cell death-1 (PD-1) monoclonal antibody used as an immune checkpoint inhibitor, is commonly employed for its anti-tumor effects against various types of malignant tumors. However, its administration is complicated by immune-related adverse events (irAEs), including pneumonitis.

**Case presentation:**

We present a case series of four patients with malignant melanoma, non-small cell lung cancer, and hypopharyngeal carcinoma who demonstrated pneumonitis induced by nivolumab, and further review clinicopathological characteristics of these patients in comparison with those of previously reported patients with nivolumab-induced pneumonitis. In our series, 20% of patients who were treated with nivolumab developed pneumonitis, all of which occurred approximately 2 weeks after the initiation of nivolumab treatment. Prompt recognition of the nivolumab-induced pneumonitis allowed for successful resolution. Computed tomography scan images of the patients demonstrated predominantly cryptogenic organizing pneumonia patterns. All patients were males, who had been heavily treated with antitumor drugs prior to nivolumab.

**Conclusions:**

Our case series showed that nivolumab had a high incidence of drug-induced pneumonitis with early onset, supporting the need for renewed attention to nivolumab-induced pneumonitis, particularly in patients with a history of heavy antitumor treatments.

## Background

Nivolumab—an immune checkpoint inhibitor—is the humanized IgG4 monoclonal antibody that targets the programmed cell death 1 (PD-1) protein, which is an immunoinhibitory receptor expressed on T cells [1]. It promotes immune responses by blocking the binding of PD-1 and its ligands, PD-L1 and PD-L2 [1]. Nivolumab produces durable objective responses in patients with malignant tumors with a favorable safety profile, and is currently used for the treatment of patients with malignant melanoma (MM), non-small cell lung cancer (NSCLC), renal cell carcinoma, Hodgkin’s lymphoma, and head and neck cancer (HNC) [2–7].

Considering the paradigm shift towards alternative therapeutic strategies for the treatment of the above-mentioned tumors, immune checkpoint inhibitors are known to cause a variety of unique toxicities that have not been well characterized. These effects termed as “immune-related adverse events (irAEs)” that include endocrine dysfunction, neurological disorder, hepatitis, nephritis, skin toxicity, cardiac insufficiency, colitis, and pneumonitis. Of these irAEs, pneumonitis is a relatively uncommon, but potentially life-threatening adverse event. A previous meta-analysis revealed that the incidence of pneumonitis associated with a monotherapy of PD-1 inhibitors, either nivolumab or another inhibitor, pembrolizumab, was 2.7% for all-grade and 0.8% for grade ≥ 3 [8]. Furthermore, this report showed that pneumonitis related to nivolumab monotherapy occurred in 4.1% (1.4–8.5%) of patients with NSCLC with all grades and 1.7% (0–3.4%) with ≥ grade 3 toxicity, while pneumonitis occurred in 1.5% (0–1.9%) of patients with MM at all grades and 0.1% (0–0.3%) with ≥ grade 3 toxicity. Although the prevalence of nivolumab-induced pneumonitis varies across tumor types, particular caution should be taken regarding this issue in NSCLC patients. A recent retrospective study demonstrated diverse computed tomography (CT) image patterns and clinical courses of nivolumab-induced pneumonitis, including predominant cryptogenic organizing pneumonia (COP) pattern, median onset time of 2.6 months, and successful treatment with corticosteroids for most patients [9].

Of 20 patients with malignant tumor who were treated with nivolumab from October 2014 to July 2017 at Tokyo Medical University Hachioji Medical Center, we encountered four cases of nivolumab-induced pneumonitis: two patients with NSCLC, one patient each with MM and HNC. Interestingly, these patients showed the different clinical characteristics of pneumonitis from those reported previously. In our cases, nivolumab-induced pneumonitis was identified as earlier onset and higher incidence, and affected patients had a history of heavy anti-tumor treatment [8–11]. In order to renew clinicians’ attention regarding pneumonitis, this report presented CT image findings and clinical course of pneumonitis following nivolumab monotherapy in four cases involving NSCLC, MM, and HNC, respectively.

## Case presentation

### Patient 1

**Fig. 1:**
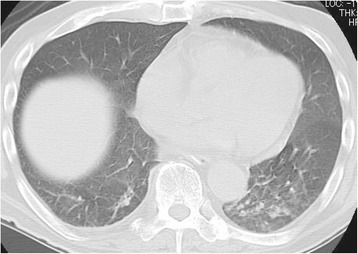
Computed tomography image of nivolumab-induced pneumonitis in Case 1. On the 17th day of nivolumab treatment, non-segmental ground-glass opacities developed at lower lobes of the bilateral lungs

A 71-year-old man with no history of smoking was admitted for a tumor of the left hallux, which was subsequently surgically resected and diagnosed as MM (pT1bN0M0). Although the patient was treated with interferon-β, the tumor recurred as multiple lymphatic metastases after 2 years, and additional chemotherapy with dacarbazine was initiated for treating the recurrence. The patient developed metastases to brain, lung, and kidney 3 years after treatment with dacarbazine. Treatment with nivolumab (2 mg/kg) was initiated for MM (rT0N2bM1c) 4 days after whole brain irradiation (total 30 Gy). On the 9th day of the initial nivolumab administration, fever developed and the patient subsequently presented with dyspnea on exertion. CT scans on the 17th day showed non-segmental ground-glass opacities (GGOs) in the lower lobes of both the lungs (Fig. 1). The possibility of infection was negated after assessment of the patient’s urinary antigen test, sputum and blood cultures, as well as tests for viral antibodies and influenza antigen. The patient was thus diagnosed with grade 1 nivolumab-induced pneumonitis with a non-specific interstitial pneumonia (NSIP) pattern according to the common terminology criteria for adverse events (CTCAE) version 4.0. Owing to the absence of respiratory failure, cessation of nivolumab treatment without additional therapies such as corticosteroids resulted in the improvement of the pneumonitis and disappearance of symptoms.

### Patient 2

**Fig. 2:**
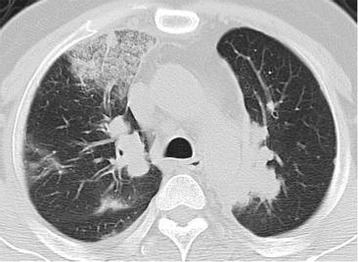
Computed tomography image of nivolumab-induced pneumonitis in Case 2. Multiple consolidations and ground-glass opacities developed at both lungs

A 46-year-old man with no history of smoking was admitted for left pleural effusion and a mass lesion at the left upper lobe of the lung. Pathological results of thoracoscopic pleural biopsy identified adenocarcinoma, and the patient was clinically diagnosed with stage IV pulmonary adenocarcinoma (cT2aN2M1a). The tumor harbored neither the epidermal growth factor receptor (*EGFR*) gene mutation nor the anaplastic lymphoma kinase (*ALK*) gene rearrangement. The patient was treated with 4 cycles of combination chemotherapy with cisplatin, pemetrexed, and bevacizumab, followed by 8 cycles of maintenance treatment with bevacizumab. Disease progression necessitated changing the regimen to docetaxel monotherapy, which was subsequently aborted due to grade 1 anaphylaxis. The patient then received combination chemotherapy with carboplatin and tegafur/gimeracil/oteracil; however, subsequent metastases to his thoracic spine occurred after 4 cycles of the treatment. As a fourth-line treatment, nivolumab (3 mg/kg) was initiated for lung cancer (rT2aN2M1b) 2 months after termination of the prior treatment. On the 13th day of the initial nivolumab administration, the patient had fever and dyspnea, and the CT results showed multiple consolidations and GGOs in both lungs (Fig. 2). Pneumonia from infection was ruled out by negative results of sputum cultures, urinary antigen tests, β-D glucan, and viral antibody tests; however, no bronchoscopy was performed due to pneumonitis-induced respiratory failure of the patient. These results along with the CT images and the clinical course concluded in a diagnosis of grade 3 nivolumab-induced pneumonitis with a COP pattern. Thereafter, methylprednisolone pulse therapy (1000 mg daily for 3 days) was initiated followed by prednisolone treatment (60 mg daily); this regimen resulted in the regression of pulmonary opacities and symptoms. Subsequently, the patient’s prednisolone dosage was decreased.

### Patient 3

**Fig. 3:**
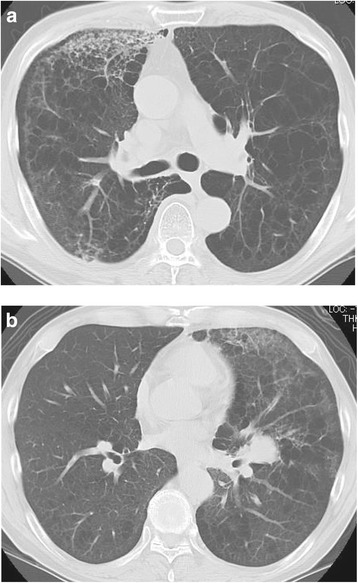
Computed tomography images of nivolumab-induced pneumonitis in Case 3. **a** Non-segmental ground-glass opacities and consolidations were observed in a predominantly subpleural distribution at both lungs on the 14th day of the initial nivolumab treatment. **b** After the predominant lesion at the right lung in the initial disease episode was reduced, the similar opacity was newly observed at the left lung in the time of relapse

A 59-year-old man with a heavy smoking history (52.5 pack-years) was admitted for right back pain. CT findings showed masses at the right upper lobe and the left lower lobe of the lung, along with right pleural effusion, right mediastinal lymphadenopathies, and marked low attenuation areas. CT-guided fine-needle biopsy pathologically identified adenocarcinoma, and the patient was diagnosed with stage IV pulmonary adenocarcinoma (cT3N2M1a) without the *EGFR* mutation or the *ALK* rearrangement. The patient underwent 6 lines of chemotherapeutic regimens: 4 cycles of 1st-line chemotherapy with cisplatin, pemetrexed, and bevacizumab, followed by 4 cycles of maintenance treatment with pemetrexed and bevacizumab; 4 cycles of 2nd-line docetaxel monotherapy; 4 cycles of 3rd-line chemotherapy with carboplatin and nanoparticle albumin-bound paclitaxel; 4th-line treatment with erlotinib for 6 months; 4 cycles of the 5th-line chemotherapy with gemcitabine and vinorelbine; and 6th-line treatment with tegafur/gimeracil/oteracil monotherapy for 5 months. Furthermore, a 7th-line treatment with nivolumab (3 mg/kg) was initiated due to deterioration of the patient’s lung cancer (rT3N3M1b) 10 months after palliative irradiation for tumor invasion of the chest wall (16 fractions to a total of 40 Gy) and 7 months after stereotactic radiosurgery for metastatic brain tumors. On the 14th day of the initial nivolumab treatment, the patient had developed dyspnea, and new non-segmental GGOs with a predominant subpleural distribution in both lungs, as determined via CT scans (Fig. 3a). Based on these imaging findings, the clinical course, and negative results of sputum cultures, urinary antigen tests, the β-D glucan value, and viral antibody tests, this patient was diagnosed with grade 3 nivolumab-induced pneumonitis with a COP pattern. Prednisolone treatment (30 mg daily) was initiated, which was subsequently decreased because of improvement of the patient’s symptoms. However, the pneumonitis was exacerbated when prednisolone was administered at a dose of 5 mg daily. While the predominant lesion in the right lung was reduced in the initial disease episode, a new lesion with similar opacity was observed in the left lung at the time of relapse (Fig. 3b). After the dose of prednisolone was raised to 60 mg daily, the pneumonitis clearly regressed, allowing for the tapering of prednisolone dose again.

### Patient 4

**Fig. 4:**
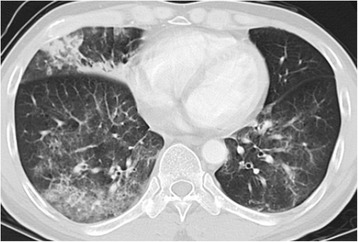
Computed tomography images of nivolumab-induced pneumonitis in Case 4. Multiple consolidations and ground-glass opacities developed at both lungs

A 58-year-old man with a heavy smoking history (52.5 pack-years) was admitted for left cervical pain. After biopsy of lesions in the pyriform sinus of the hypopharynx, the patient was diagnosed with a hypopharyngeal squamous cell carcinoma (cT3N2cM0). Following induction chemotherapy with cisplatin, docetaxel, and 5-FU, the patient underwent seven cycles of the concurrent cetuximab and radiation therapy, and experienced complete response to treatment. He received 9 cycles of combination chemotherapy with nedaplatin, cetuximab, and tegafur/gimeracil/oteracil because of a recurrence in the left deep cervical lymph node after seven months. Thereafter, 49 cycles of combination chemotherapy with paclitaxel and cetuximab was initiated, but resulted in progressive disease from enlarged metastasis of left deep cervical lymph node. On the 4th day of the 2nd cycle of nivolumab treatment (3 mg/kg), the patient exhibited malaise and exertional dyspnea symptoms, and CT results showed multiple GGOs and consolidations in both lungs (Fig. 4). Based on the imaging results and clinical course, he was diagnosed with respiratory insufficiency caused by nivolumab-induced pneumonitis (grade 3). Sputum cultures, urinary antigen tests, the β-D glucan value, and viral antibody tests were all negative. Along with inhalation of oxygen, the patient was subjected to methylprednisolone pulse therapy (1000 mg daily for 3 days) followed by prednisolone treatment (30 mg daily), which resulted in the regression of pulmonary lesions, and facilitated the tapering of prednisolone dose.

## Discussion and conclusions

Treatment with nivolumab induces a variety of adverse events, including irAEs which can sometimes be serious or fatal, albeit infrequent. Among them, pneumonitis is one of the most life-threatening adverse events. Four patients discussed in the present report were diagnosed with nivolumab-induced pneumonitis, mainly based on the image findings and clinical courses. All four patients had no pre-existing interstitial pneumonia as a potential risk factor for drug-induced pneumonitis. Furthermore, in statistical analyses (data not shown), there were no significant differences in clinicopathological characteristics between patients with and without nivolumab-induced pneumonitis.

In contrast, these four patients had some characteristics that were different from patients with nivolumab-induced pneumonitis in the previously published reports (Table 1). First, the meta-analysis performed by Nishino et al. results revealed that the incidence of the pneumonitis was 2.7% for nivolumab monotherapy and pneumonitis-related death was relatively rare [8]. Another study reported that the incidence of pneumonitis associated with nivolumab monotherapy was 2.9% and 11.8% for a combination therapy with nivolumab and other immune checkpoint inhibitors [9]. Meanwhile, four (20%) out of 20 patients, who underwent nivolumab treatment in our institute developed pneumonitis (22% for NSCLC, 17% for MM, and 25% for HNC); although the number of cases included in this study was small, a strikingly larger proportion of patients developed nivolumab-induced pneumonitis in our study than that reported previously. In a previous study on Japanese patients, male gender and smoking history were suggested to be potential risk factors for nivolumab-related pneumonitis [11]. In our cases, all the patients who developed pneumonitis were Japanese males. As the previous study suggests higher incidence of drug-induced pneumonitis in Japanese patients, the ethnicity also may impact on nivolumab-induced pneumonitis [12]. Another study reported that all lung cancer patients were administered 3–5 lines of preceding systemic therapy till the onset of nivolumab-induced pneumonitis, although no significant differences in trends regarding gender were observed [9]. In our institute, all patients were also heavily treated with pharmaceutical or radiation therapy before nivolumab treatment, and it is possible that accumulated treatment histories in male patients may be associated with higher incidence of pneumonitis. Second, the median time from initiation of nivolumab treatment to onset of pneumonitis in our cases was 15 days (12.5 days in NSCLC, 17 days in MM, and 18 days in HNC), whereas the median time to onset of pneumonitis in previous reports was 1.2 months in NSCLC patients, 3.6 months in MM patients, and 40 days in Japanese patients with NSCLC [9–11]. Furthermore, Naidoo et al. reported that time to onset of pneumonitis induced by anti-PD-1/PD-L1 antibodies ranged from 9 days to 19.2 months [13]. The reason for the earlier onset in our cases remains unknown. Pneumonitis in our report was promptly ameliorated in all patients, who were non-fatal. In this context, the early onset in our cases may be a result of an early detection of the patients’ symptoms, although it must be mentioned that this study comprised of only four cases.

**Table 1:** Patient characteristics

Case no.	Age	Sex	PS	Tumor type	Stage	Smoking history	Treatment line	Treatment cycle	Diagnosis to treatments	Initiation of nivolumab to onset of pneumonitis	Radiographic pattern	Distribution	Major lesion	CTCAE grade pneumonitis	Treatment for pneumonitis	Outcome of penumonitis	Survival from initiations of nivolumab
1	71	Male	1	MM	IV	No	2nd	1	61 months	17 days	NSIP	Bilateral	Lt lower lobe	1	None	Remission	158 days
2	46	Male	1	NSCLC	IV	No	4th	1	23 months	12 days	COP	Bilateral	Rt upper lobe	3	Corticosteroids	Remission	109 days
3	58	Male	1	NSCLC	IV	54	7th	1	43 months	13 days	COP	Bilateral	Rt upper lobe	3	Corticosteroids	Remission	172 days
4	58	Male	1	HNC	Iva	52.5	5th	2	36 months	18 days	COP	Right	Right	3	Corticosteroids	Remission	156 days

*PS* performance status, *MM* malignant melanoma, *NSCLC* non-small cell lung cancer, *HNC* head and neck cancer, *CTCAE* common terminology criteria for adverse events V4.0, *NSIP* nonspecific interstitial pneumonia, *COP* cryotpgenic organizing pneumonia, *Lt* left, *Rt* right

Importantly, the pneumonitis induced by one drug can present various disease and image patterns, and conversely, numerous drugs have been reported to develop the pneumonitis with a similar pattern. Furthermore, previous retrospective studies and meta-analyses of nivolumab-induced pneumonitis have also shown a similarly wide range of image patterns, with COP pattern being the most common [8, 9]. Interstitial lung disease with COP and NSIP patterns commonly has a better response to corticosteroids than those with usual interstitial pneumonia (UIP)/idiopathic pulmonary fibrosis (IPF) pattern [14, 15]. Image findings of COP and NSIP patterns may reflect a favorable outcome in the pneumonitis of our cases. In fact, many cases of nivolumab-induced pneumonitis were also responsive to corticosteroids, which may be attributed to predominant COP pattern in these cases. However, nivolumab-induced pneumonitis with COP or NSIP pattern have also been shown to sometimes result in diffuse alveolar damage, further leading to lethality [11, 13, 16].

Although currently no optimal management guideline exists for the management of pneumonitis, considering the widespread use of immune checkpoint inhibitors, it is necessary to take particular caution to avoid or manage drug-related pneumonitis. Our case series showed that nivolumab had a high incidence of drug-induced pneumonitis with early onset, supporting the need for renewed attention to nivolumab-induced pneumonitis, particularly in patients with a history of heavy anti-tumor treatment.

## Abbreviations

ALK: Anaplastic lymphoma kinase
COP: Cryptogenic organizing pneumonia
CT: Computed tomography
CTCAE: Common terminology criteria for adverse events
EGFR: Epidermal growth factor receptor
GGOs: Ground-glass opacities
HNC: Head and neck cancer
IPF: Idiopathic pulmonary fibrosis
irAE: Immune-related adverse events
IRB: Institutional review board
MM: Malignant melanoma
NSCLC: Non-small cell lung cancer
NSIP: Non-specific interstitial pneumonia
PD-1: Anti-programmed cell death-1
UIP: Usual interstitial pneumonia
